# Physical activity, lifestyle, sociocultural factors and prevalence of excess weight gain among postmenopausal women: A cross-sectional study

**DOI:** 10.1177/17455057231184508

**Published:** 2023-12-15

**Authors:** Isaac Mensah Bonsu, Corlia Brandt, Adedayo Tunde Ajidahun, Moses Omoniyi, Hellen Myezwa

**Affiliations:** 1 Department of Physiotherapy, School of Therapeutic Sciences, University of the Witwatersrand, Johannesburg, South Africa; 2 Department of Physiotherapy and Sports Science, Faculty of Allied Health Sciences, College of Health Sciences, Kwame Nkrumah University of Science and Technology, Kumasi, Ghana

**Keywords:** Ghanaian, physical activities, postmenopausal women, sociocultural aspect, weight-gained

## Abstract

**Background:**

Most women experienced postmenopausal symptoms during the menopause transition, and they are a common reason for seeking medical attention and lifestyle modification during this phase of life.

**Objective:**

This study assessed the physical activity, lifestyle, and sociocultural levels-associated prevalence of excess weight (overweight and obesity) among postmenopausal women (PW) in Bono-East (Techiman) region, Ghana.

**Design:**

This is cross-sectional study.

**Method:**

This was a cross-sectional study conducted at Bono-East regional capital, Techiman in Ghana over 5 months. Self-administered questionnaires were used to obtain sociodemographic data, physical activity, lifestyle, and sociocultural associated prevalence of excess weight. Anthropometric indices including ((body mass index (BMI), waist-to-height ratio (WHtR), and waist-to-hip ratio (WHR)) were assessed.

**Results:**

A total of 393 postmenopausal women with a mean age of 60.09 ± 6.24 years participated in the study. When reporting prevalence, obesity, and overweight were distinguished. Using the anthropometric parameters (BMI, WHtR, and WHR) as measured for being overweight, the prevalent rates were 35.7%, 21.7%, and 9.0% respectively. Also, when using obesity, the prevalent rate was 37.8%, 70.1%, and 82.0% using BMI, WHtR, and WHR, respectively. Over 55 (55.2%) of the study participants engaged in moderate physical activities, 23.1% were low and 21.7% with high physical activities. Housewives and unemployed participants are shown to have a higher risk of gaining weight (obese) especially when assessed with WHR and WHtR. Most participants responded that cultural beliefs prevent them from losing weight. Most participants had a poor attitude toward their eating habits.

**Conclusion:**

According to the findings, postmenopausal Ghanaian women showed a high level of obesity and a moderate level of being overweight. The cultural perception of beauty influences Ghanaian postmenopausal women’s physical activity level and dietary habits.

## Introduction

Menopause is a cycle that happens spontaneously or is medically induced in women to mark the end of their ability to reproduce.^1^ In comparison to medically induced menopause, natural menopause is a progressive cycle with permanent cessation of menstruation which is determined 12 months after the last menstrual period, which occurs in most women between 47 and 55 years of age.^2^ Average age of menopause is 51 years.^3^ However, women in Ghana experience menopause at a mean age of 48 years.^4^ For women aged 55–65 years, weight gain is one of their major health concerns during this period.^5^ Menopausal women frequently experience weight gain^6^ and several observational studies indicate that weight gain is a major concern for menopausal women during the transition.^7–10^ According to literature, women gain an average of 0.4 kg per year throughout the menopausal transition.^11^ Although average weight gain varies considerably, 20% of women during this transitional period gain 4.5 kg or more.^11^ It has been demonstrated that the increase in body weight is related to aging and a decline in energy expenditure.^12,13^ Notwithstanding, increased visceral and abdominal subcutaneous adipose tissue deposition is associated with menopause.^6^ This central adiposity is associated with increased cardiovascular and metabolic diseases, as well as decreased physical activity and poorer quality of life.^12^

The risks of excess body weight and related comorbidities are influenced by diet, physical inactivity, perceptions of health, and cultural beliefs.^14^ The World Health Organization^15^ claims that lack of physical activity, for example, has been recognized as the fourth major cause of global death, accounting for 27% of diabetes cases, and about 30% of ischemic heart disease cases.

A study conducted by Boakye et al.^16^ indicated that the overall prevalence of the certain non-communicable diseases was 26.7% (CI = 0.23–0.31), of which hypertension (22.7%) was the most prevalent. The study further concluded that more than half (54%) were physically inactive and the odds of developing NCDs were higher in females.^16^ If current trends continue, it is projected that NCDs would exceed all other causes of mortality in Ghana by 2030 and in Africa by 2035.^17^ Many non-communicable diseases, such as cardiovascular disease and diabetes, have also been linked to sedentary behavior.^18,19^

As the burden of excess weight increases in Africa,^20,21^ high social and cultural valuation of large body size or fatness have considerably contributed to the growing prevalence of overweight and obesity.^22,23^ For instance, Rguibi and Belahsen^24^ highlighted the role of sociocultural factors in the maintenance of traditional values about the desirability of body weight. The same study revealed that most Saharawi women desired to gain weight during their life because of its association with beauty.^24^ Okop et al.^25^ explored the obesity risk awareness, perception of body size and willingness to lose body weight in adults aged 35–70 years. They concluded that the satisfaction of large size as attractive and normal indicate a considerable challenge to obesity prevention.^25^ Within Ghanaian cultural context, studies have shown that Ghanaian women are comfortable with being overweight, albeit not severely,^26,27^ and in Africa.^28^ Women who become overweight/obese are unlikely to be displeased with their weight status in a cultural environment that sanctioned large body size.^29^ These could limit attempts to follow interventions against excessive fat accumulation. However, in a society where “plumpness” is traditionally valued, understanding the sociocultural associated effect of body size/weight would inform interventions against obesity as a problem.^30^

Numerous studies have assessed excess weight problems solely from a nutrition and health perspective;^31^ however, there is a dearth of literature evaluating the holistic multifaceted factors involving physical activity, lifestyle, and sociocultural influence in weight gain among postmenopausal women in Ghana. This cross-sectional study aimed to evaluate the levels of physical activity, lifestyle, sociocultural factors, and prevalence of excess weight gain among postmenopausal women in Ghana.

## Methods and materials

### Study design and setting

The cross-sectional study was conducted between October 2019 and February 2020. Anthropometric measurements were performed on eligible women of selected women organizations and fellowships, mosques, churches, and outpatient departments (OPD) of the hospitals at Bono-East regional capital, Techiman in Ghana. Participation in the study was voluntary and participants also consented by appending their signatures on the consent forms. Authors followed STROBE Guidelines when preparing the manuscript.

### Participants, sampling and sample size

The study population was selected by stratifying the country Ghana into three geographical zones: northern, middle and southern zones. Sequentially, a zone, region, and district were selected by simple random sampling. Consecutive convenience sampling was used to select churches, mosques, hospitals, women’s fellowships, and women’s organizations from the district. The study site is the most diverse and well-populated city in the Bono-East region. Site selection was based on the high number of participants who matched the study’s selection criteria and the similarities between the sample and the general population. The sample frame comprised postmenopausal women in the Bono-East region, aged 45 years or older using the 2010 population and housing census in Ghana.^32^ A sample size of 358 was estimated using the sample size (Formula 1) for cross-sectional studies in an unknown population.^33^ The final sample size was 393 with a 10% fall-out rate was considered. The calculation was based on the prevalence of obesity among postmenopausal women in Ghana (37.1%, *p* = 0.37),^34^ a margin of error set at 0.05 and a 95% confidence interval.

Formula 1: Sample size formula for cross-sectional studies

(Z2)2P(1−P)d^2

*N* is the sample size, *Z* is the statistic corresponding to level of confidence, *P* is expected prevalence, and *d* is margin of error.

### Data collection

Self-administered questionnaires included sociodemographics, physical activity, lifestyle, sociocultural factors, and anthropometric measurements.

### Physical activity assessment

Physical activity level among participants was assessed through the International Physical Activity Questionnaire (IPAQ) Short Form. Participants indicate time and the number of days within the previous 7 days they spent walking, doing a moderate-intensity activity and/or doing vigorous-intensity activities.^35^ Globally, this questionnaire is used to evaluate physical activity (indicated as low moderate and high) over a period of 7 days.^35^

### Lifestyle factors

The questionnaire included different questions related to lifestyle, including health-related habits (alcohol consumption and smoking status), leisure time activity and time spent watching television. According to smoking status, participants were classified as never smokers, past smokers, or current smokers. Marital status was categorized as single, married, widowed or divorced. Participants were asked to describe their lifestyle as very sedentary, sedentary, moderately active, or very active. Seven specific questions were designed to assess the frequency of certain dietary habits during a usual week based on 7-day 24-h dietary recalls. The participants were asked to state how often they consume breakfast, lunch and dinner, water and high-content foods (carbohydrate, protein, fat, vegetables). The high-content carbohydrate foods in this regard included some examples from Ghanaian local foods, such as “banku, fufu, kenkey, tuozafi and yam.” The questions covered some healthy and unhealthy dietary habits. The beverages were categorized into four levels of intake: fruit juice, mineral water, soft drink, and alcoholic drink.

### Sociocultural factors

The questions on sociocultural factors were designed to assess how traditional customs and cultural background influence the body weight of the participants. Social variables including employment status were assessed. Participants were asked about society’s influences on their body weight and whether cultural background and beliefs may prevent them from losing weight.

### Anthropometric measurements

Participants’ body weight and height were determined to the nearest 0.5 kg and 0.5 cm, respectively, using a Seca weighing scale (Seca weighing scale: Seca Gmbh & Co. KG; 22,089 Hamburg, Germany; Model: 8741321009; designed in China) and stadiometer (Ghaziabad, Scorpia India Medicare Pvt. Ltd.—ID: 4076377262). Body mass index was assessed as the ratio of body weight to height squared after ensuring standardized method measurement, BMI > 25.0 kg/m^2^ is considered excess weight.^36^ The waist circumference (WC) was measured based on World Health Organization guidelines to the nearest 0.1 cm. It was measured between the lower border of the rib cage and the iliac crest using a non-stretchable fiberglass measuring tape. Participants with a WC 80 cm and above were considered as having abdominal obesity, as defined by the International Diabetes Federation.^37^ To define the waist-to-hip ratio (WHR), WC was divided by the hip circumference, measured at the widest location of the hips. According to the WHO reference values, results higher than 0.85 were considered a benchmark for metabolic syndrome.^38^ We calculated WHtR by dividing WC (cm) by the measured height (cm) and WHtR ⩾ 0.5 is adopted as excess weight.^39^ Body mass index (BMI), waist-to-hip ratio (WHR), and waist-to-height ratio (WHtR) were calculated and categorized according to the WHO reference values.^40^

### Statistical analyses

Data were analyzed using IBM SPSS (version 25.0). All variables were summarized using descriptive statistics, namely frequencies, percentages for data in graphic presentations, mean values, and standard deviation. Odd ratio and confidence interval were used to assess the association between demographic variables and the various indexes of weight gain. Statistical significance was set at p < 0.05 for all comparisons. To determine whether continuous variables had a normal distribution, a Shapiro–Wilk test was used. Variables that were continuous and normally distributed were presented as mean ± standard deviation (SD). Missing data were treated as missing.

## Results

The age range of the 393 postmenopausal women enrolled in this study was from 45 to 80 years with a mean age of 60.09 ± 6.24 years. The highest proportion was in the age band 51–60 years contributing 48.8% of all participants. The sociocultural information showed that most of the participants 45.2% (*n* = 164) were employed and less than half of the participants (41.6%, *n* = 155) considered themselves to be overweight. Cultural background and beliefs were considered by 50.8% (*n* = 188) of the total cohort as preventing them from losing weight. Half of the participants responded that society views their current weight as attractive (Table 1).

**Table 1.** Demographic, anthropometric variables, and sociocultural characteristics of the participants (*N* = 393).

Demographics	Number of respondent, *n* (%)
Age in years, mean (SD)	60.09 (6.24)
Age group (years),^a^ *n* = 369 (%)	
⩽50	24 (6.5)
51–60	180 (48.8)
61–70	150 (40.7)
>70	15 (4.2)
BMI (mean ± SD)	73.50 + 13.870
WHR (mean ± SD)	0.91 + 0.088
WHtR (mean ± SD)	0.62 + 0.140
Employment status,^a^ *n* = 363 (%)	
Employed	164 (45.2)
Retired	67 (18.4)
Housewives	24 (6.6)
Unemployed	102 (28.1)
Others	6 (1.7)
Are you overweight,^a^ *n* = 373 (%)	
Yes	155 (41.6)
No	130 (34.8)
Not sure	88 (23.6)
Perception and understanding of overweight,^a^ *n* = 365 (%)	
Absence of disease	82 (22.4)
Presence of disease	159 (43.6)
Not sure	124 (34.0)
In your customs, do you think an overweight woman will be recognized,^a^ *n* = 372 (%)	
Yes	193 (51.9)
No	118 (31.7)
Not sure	61 (16.4)
Cultural background and beliefs may prevent me from losing weight,^a^ *n* = 370 (%)	
Yes	188 (50.8)
No	182 (49.2)
Society’s view and perception of current body weight,^a^ *n* = 372 (%)	
Yes	186 (50.0)
No	121 (32.5)
Not sure	65 (17.5)

SD: standard deviation; BMI: body mass index; WHR: waist-to-hip ratio; WHtR: waist-to-height ratio.

a Missing data.

**Figure 1.**
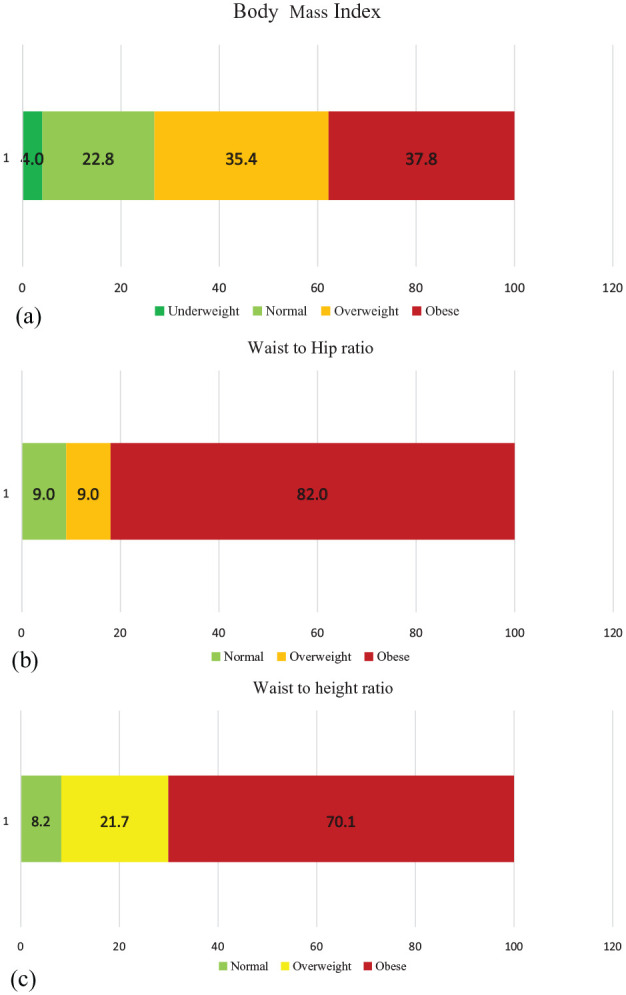
(a) Prevalence of obesity among study participants stratified by body mass index categories. (b) Prevalence of obesity among study participants stratified by waist to hip ratio categories. (c) Prevalence of obesity among study participants stratified by waist to height ratio categories.

Figure 1 shows distribution of obesity among study participants. From Figure 1(a), 37.8% were obese, 35.4% were overweight, 22.8% normal, and 4.0% underweight. According to WHR assessment in Figure 1(b), 82.0% were obese, 9.0% overweight and normal respectively. In Figure 1(c), 70.1% were obese and 8.2% were normal using WHtR.

### Physical activity

**Figure 2.**
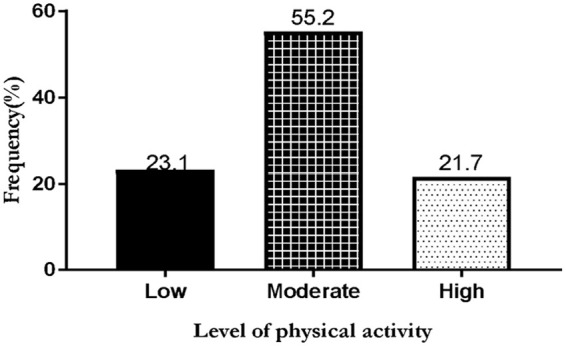
Prevalence of physical activity level among study participants.

Table 2 shows vigorous activities like heavy lifting, digging, aerobics, and fast bicycling as activities in which study participants engaged in. They often participate in vigorous physical activity from 1 to 2 times per week (mean value of 2.1) for at least 1 to 10 min (mean value of 1.3). During the last 7 days, participants responded that they had 1 to 2 days per week of moderate physical activities (mean value of 1.3) with a duration of 1 to 20 min per day (mean value of 1.5). Study participants walked 1 to 3 days per week (mean value of 2.4) and on a day they spend walking 1 to 20 min per day (mean value of 1.5). Participants spend about 30 to 60 min per day sitting on weekdays. From Figure 2, 55.2% of the study participants engaged in moderate physical activities 23.1% in low-level activities, and 21.7% in high physical activity.

**Table 2.** Assessment of physical activity pattern among study participants, *N* (393).

Physical activity	Mean (*n*)	SE	Minimum	Maximum
Vigorous activities				
How often during the last 7 days	2.1	0.05	1	3
Duration of the activities	1.3	0.05	0	3
Moderate activities				
How often during the last 7 days	1.3	0.03	0	2
Duration of the activities	1.5	0.04	0	3
Time spent walking				
Walking/day at least 10 mins	2.4	0.03	1	3
Duration of walking on a day	1.5	0.04	0	3
Time spent sitting				
Time sitting/day	2.1	0.04	0	3
Hours spend watching TV	3.3	0.04	1	4

SE: standard error.

### Lifestyle factors

Most of the participants (68.7%) reported eating three times a day (breakfast, lunch, and dinner) and 68.3% drink between 1 and 1.5 L of water per day (Table 3). Among the total study participants, 59.7% said they typically eat more than they should for about 4–7 days.

**Table 3.** Information on diet and lifestyle among participants.

Do you usually eat breakfast, lunch and dinner every day, *n* = 371 (%)	
Yes	255 (68.7)
No	102 (27.5)
Not sure	14 (3.8)
What is your diet pattern, *n* = 368 (%)	
Different every day	126 (33.9)
Different only sometimes during a week	130 (34.9)
Different only during the weekend’s days	80 (21.5)
Same everyday	36 (9.7)
Over the past 7 days, how often do you eat high protein, carbohydrate, fat, and vegetable content food, *n* = 370 (%)	
1–3 days	137 (37.0)
4–7 days	220 (59.5)
Not sure	13 (3.5)
Which beverages do you usually drink between meals, *n* = 372 (%)	
Mineral water	247 (66.4)
Soft drinks	70 (18.8)
Alcoholic drinks	28 (7.5)
Fruit juice	27 (7.3)
Do you take beverages in all your meals, *n* = 371 (%)	
All the time	197 (530)
Sometimes	156 (42)
Not sure	18 (4.9)
Do you drink at least 1–1.5 L of water every day, *n* = 372 (%)	
Yes	254 (68.3)
No	88 (23.7)
Not sure	30 (8)
Lifestyle characteristics	
Smoking status, *n* = 373 (%)	
Never	339 (90.9)
Former	24 (6.4)
Current	10 (2.7)
Alcohol intake, *n* = 365	
Yes	113 (31.0)
No	252 (69.0)
Describe your lifestyle, *n* = 370 (%)	
Very sedentary	28 (7.6)
Sedentary	95 (25.7)
Moderately active	167 (45.1)
Very active	80 (21.6)
Hours spent watching TV, *n* = 370 (%)	
1–2 h a day	237 (64.1)
3–4 h a day	105 (28.4)
5–6 h a day	28 (1.2)
I will never lose weight due to my lifestyle, *n* = 362 (%)	
Yes	199 (55.0)
No	163 (45.0)

TV: television.

Most of the participants 90.9% (*n* = 339) reported never smoked or taken alcohol 69.0% (*n* = 252), respectively. Most of them 64.1% (*n* = 237) spend their time watching TV and slightly less than half 45.0% (*n* = 163) agreed that they gain weight due to their lifestyle. The results are shown in Table 3.

Odds ratio was used to determine the association between sociocultural factors and three predictors of obesity (BMI, WHR, and WHtR) among study participants (Table 4). Participants who were employed, housewives, unemployed and other statuses of employment were all at lower risk of BMI-obesity (i.e. OR < 1) compared to the retirees. The unemployed had the highest risks of WHR-obesity (1.6) and WHtR-obesity (2.4). Participants who considered themselves overweight were at higher risk for BMI-obesity, WHR-obesity and WHtR-obesity compared to those who reported not being overweight. Participants who stated that their cultural background and beliefs could prevent them from losing weight were 1.4 times at risk of BMI obesity compared to those who thought otherwise. Participants with high physical activity generally had higher odds of obesity.

**Table 4.** Odds ratio of sociocultural risk factors for obesity (using BMI, WHR, and WHtR) among study participants.

Sociocultural characteristics	BMI-obesity aOR (95%CI)	WHR-obesity aOR (95%CI)	WHtR-obesity aOR (95%CI)
Employment status			
Employed	1.0 (0.5–2.1)	1.1 (0.4–3.0)	0.8 (0.3–2.4)
Retired	1	1	1
Housewives	0.6 (0.2–1.8)	–	1.9 (0.2–17.9)
Unemployed	0.7 (0.3–1.7)	1.6 (0.5–5.6)	2.4 (0.6–10.0)
Others	0.6 (0.1–3.6)	0.6 (0.1–6.4)	0.6 (0.1–6.4)
Are you overweight?			
Yes	1.9 (1.0–3.6)	1.0 (0.4–2.8)	1.3 (0.4–3.6)
No	1	1	1
Not sure	0.7 (0.3–1.4)	0.5 (0.2–1.6)	0.7 (0.2–2.2)
Understanding of overweight			
Absence of disease	1	1	1
Presence of disease	0.6 (0.3–1.2)	1.2 (0.4–3.5)	0.9 (0.3–2.6)
Not sure	0.9 (0.4–1.9)	1.3 (0.4–4.3)	0.9 (0.3–3.3)
Overweight women recognized?			
Yes	1	1	1
No	1.5 (0.8–2.9)	1.3 (0.5–3.5)	0.9 (0.3–2.6)
Not sure	1.6 (0.8–3.4)	0.7 (0.2–2.0)	0.5 (0.2–1.4)
Cultural beliefs preventing losing weight			
Yes	1.4 (0.8–2.5)	0.6 (0.3–1.6)	0.6 (0.2–1.6)
No	1	1	1
Societal view on your current weight			
Attractive	0.7 (0.4–1.3)	0.9 (0.3–2.1)	0.8 (0.3–2.0)
Not attractive	1	1	1
Not sure	0.3 (0.2–0.8)*	1.3 (0.3–5.5)	0.8 (0.2–3.2)
Physical activity			
Low	1.0 (0.5–2.1)	0.7 (0.2–2.7)	0.9 (0.2–3.3)
Moderate	1.0 (0.5–2.0)	0.5 (0.2–1.7)	0.8 (0.3–2.4)
High	1	1	1

BMI: body mass index; WHR: waist to hip ratio; WHtR: waist–to–height ratio; aOR: adjusted odds ratio; CI: confidence interval.

* p-value < 0.05 = statistically significant.

## Discussion

This study assessed physical activity, lifestyle, sociocultural factors and the prevalence of excess weight gain among postmenopausal women. Our results raise an important issue, namely, that a high number of the participants (50.8%, *n* = 188) believe that cultural backgrounds encourage postmenopausal women to gain weight.

According to the findings, excess weight is viewed as attractive and symbolizes culturally appropriate body size despite its health implications. From the literature, all intervention focuses on nutrition and lifestyle modification and have not emphasized the inclusion of cultural perception which in fact influence behavior.^41^ As such the approach and measures used in managing this global epidemic which is a threat to public health need review.^29,42^ As a first step, a comprehensive program to manage excess weight and its health impact are essential. Programs, interventions, and studies focusing on the management of excess body weight must recognize the perception of society and cultural sentiment on body weight. Excluding these has the potential to underestimate the accurate effect of cultural beliefs, society’s view, and perception of body weight. Similar results have been found,^26,30,43–45^ in a study by Rguibi and Belahsen,^24^ stating that Moroccan Saharawi women practice fattening rituals to induce weight gain. In Africa, cultural belief in body image has been associated with obesity, particularly among black African women who were dissatisfied with their current body size. They perceived larger body sizes as ideal body size.^44,45^ Physical activity is known to be effective in reducing body weight and size.^41^ Examining and use of local daily chores which themselves are physical activities may be a useful point of intervention.

Our study also indicates that unemployed postmenopausal women had the highest risk of WHR and WHtR obesity. The earlier studies of sedentary postmenopausal women with obesity and age-related disease by Kalyani et al.^46^ and Davenport et al.^47^ described the existence of declines in aerobic fitness, muscle mass, muscle strength, and bone mineral density. The decline in these components is a prevalent and important risk factor for disability and potentially mortality in individuals as they age.^46^ Preventing this risk of weight gain during this period is critical.^47^ Exercise has been demonstrated to be a simple, cost-effective means to improve these outcomes.^46^ Therefore, exercise is particularly relevant to postmenopausal women with obesity to preserve their quality of life and improve active life expectancy into old age.

Accumulating 30 min of moderate-intensity physical activity on most days is enough to provide substantial health benefits,^48^ however, our study indicates that the majority (55.2%) of the participants had 1 to 2 days per week of moderate physical activities for 1 to 20 min per day during the last 7 days. The evidence suggests that participants may not be meeting the physical activity recommendation of 150–300 min of moderate intensity per week for older adults.^49,50^ There is a need to increase the level of physical activity, particularly among postmenopausal women through promotion, education and interest in a culturally appropriate program.^51^ Developing interventions that will increase awareness and result in behavior change and increased activity is important. The World Health Organization guidelines on physical activity^49^ may need to adopt culturally appropriate activities for instance “Ampe,” which is a Ghanaian indigenous game to gain an optimal effect and impact.

Our analyses also revealed that participants engage in regular moderate physical activity such as walking. In a similar manner to walking, our study found participants walked a maximum of 3 days per week for 20 min per day. The association between duration and frequency of walking suggests that participants in this sample may not be expending sufficient energy to meet the definition of moderate-intensity physical activity of 150 min per week. A study by Ogilvie et al.,^52^ described walking as near-perfect exercise. The reason could be its popularity, familiarity and a free form of exercise that can be incorporated into everyday life and sustained into old age.^52,53^ In addition, a study suggests that walking may be influenced by physical-environmental factors and walking needs.^54^ Such results suggest that several social conditions and walking needs act as moderators before people consider deciding to walk.^54^ The findings are particularly important as our results indicate participants do not walk enough which could be due to the influences of environmental and societal conditions. However, considering that participants are more likely to be sedentary and inactive, they may find walking difficult and will find it easier to undertake their normal roles such as taking care of grandchildren and working in the market or general household chores.^55^ They will not prioritize activities such as walking and exercise. To encourage greater activity, the use of culturally appropriate activity to understand their physical activity may be useful. In addition to culturally appropriate interventions, it is important to counter the growing trend of sedentary lifestyles and poor eating habits.

Modification of a sedentary lifestyle and also eating habits have been proposed as a therapy to prevent weight gain during the postmenopausal stage.^56^ Dietary habits reported in this current study showed that more than 50% of the participants eat three times a day and on most days of the week eat more than the recommended amount. These may be the reasons for the weight gain in this current study, agreeing with the finding of studies by Rguibi and Belahsen,^24^ Amoah^43^ which revealed that eating patterns affect weight gain. A study by Petrella et al.^57^ projected that a caloric restriction associated with changes in eating behavior and constant physical activity can reduce weight gain among obese women. In addition, a summarized literature review regarding the impact of the menopause transition on body weight and body composition conducted by Douketis et al.^58^ revealed that calorie restriction alone can elicit a reduction in body weight, total body and visceral fat, similar to exercise. However, the addition of exercise, with a weight loss of more than 5%, can reduce risk factors for cardiovascular diseases, such as dyslipidemia, hypertension, and diabetes mellitus.^58^

In Ghana, fried foods, fatty foods and sugary drinks are more popular compared to four or five decades ago.^43^ Ghanaians also seem to be exercising less frequently due to changes in lifestyle and socioeconomic status that have a significant effect on physical activity. People used to walk considerable distances (sometimes miles) to work in the past. Nowadays, fewer Ghanaians walk, preferring instead to use “Pragyaya” (cheap city public transportation) or a car. Furthermore, because many more families now own a car, they are more likely to drive rather than walk. In the past, most families did not have television; those that did had only one channel (GTV), which was available from 18.00 to 22.00 hours each night.^43^ However, most households now have more than five television channels and hours of viewing a day.

These changes resulting from modernization contribute to a sedentary lifestyle and increase the likelihood of increased obesity.^55,59^ Hence, much more attention is required to promote lifestyle modification and encourage physical activity.

The results of the study provide insight into postmenopausal women’s self-reported physical activity and lifestyle status and sociocultural associated prevalence of weight gain. The results suggest both a need for physical activity program, lifestyle modification and interest in a community-based program.

### Limitation

The use of self-report methodologies to measure physical activity, lifestyle, and sociocultural outcomes was a constraint. Issues and limitations with self-reported outcomes have been raised in the literature,^60^ and these may have influenced the findings. Second, the study was not based on a representative national sample. Using recall of the number of days participants consumed food and performed physical activity on an average day may be influenced by recall bias.

## Conclusion

Our study concluded that there is a high prevalence of obesity among Ghanaian postmenopausal women and certain sociocultural risk factors increase the risk of being obese. However, most of the study participants engaged in moderate physical activities. Our findings suggest that motivating postmenopausal women to increase their physical activity and modify their lifestyle during their middle years can reduce abdominal fat during the menopause transition, which may impact positively the cardiovascular disease risk profile.

Population-based initiatives are needed to encourage physical activity and healthy eating and to conduct research to identify effective educational, behavioral, and environmental approaches to control and prevent excess weight among this population.

Successful attempts to counter sedentary lifestyles have been documented;^26,61^ however, negative social pressures such as “fat are beautiful, or prosperous” should be addressed through educational programs. Individuals and communities must be made aware of the health risks associated with obesity and being overweight. Awareness creation should be complemented by population-based health programs that emphasize the benefits of Ghanaian traditional physical activity such as cultural dances (Adowa, Borborbor, and Bamaya) and healthy eating in preventing and controlling excess weight.

## Supplemental Material

sj-docx-1-whe-10.1177_17455057231184508 – Supplemental material for Physical activity, lifestyle, sociocultural factors and prevalence of excess weight gain among postmenopausal women: A cross-sectional study Click here for additional data file. Supplemental material, sj-docx-1-whe-10.1177_17455057231184508 for Physical activity, lifestyle, sociocultural factors and prevalence of excess weight gain among postmenopausal women: A cross-sectional study by Isaac Mensah Bonsu, Corlia Brandt, Adedayo Tunde Ajidahun, Moses Omoniyi and Hellen Myezwa in Women’s Health

## Declarations

**Ethics approval and consent to participate:** Ethical approval for this study was obtained from the Human Research Ethics Committee at the University of the Witwatersrand, South Africa (Ref no. M190467). In Ghana, approval was also obtained from the Committee on Human Research, Publication and Ethics of Kwame Nkrumah University of Science and Technology, Kumasi (Ref No. 596/19). Participation was voluntary and written informed consent was obtained from each participant according to the Helsinki declaration.

**Consent for publication:** Consent for publication was obtained from the participants.

**Availability of data and materials:** The data sets analyzed in the current study are accessible from the corresponding author on fair request.
